# Angiopoietin-Like 4 (ANGPTL4) in Patients with Psoriasis, Lichen Planus and Vitiligo—A Pilot Study from the Bialystok+ Polish Longitudinal University Study

**DOI:** 10.3390/metabo12090877

**Published:** 2022-09-17

**Authors:** Julia Nowowiejska, Anna Baran, Justyna Magdalena Hermanowicz, Joanna Mikłosz, Karol Adam Kamiński, Marcin Kondraciuk, Marlena Dubatówka, Dariusz Pawlak, Iwona Flisiak

**Affiliations:** 1 Department of Dermatology and Venereology, Medical University of Bialystok, Zurawia 14 St, 15-540 Bialystok, Poland; 2 Department of Pharmacodynamics, Medical University of Bialystok, Mickiewicza 2C St, 15-540 Bialystok, Poland; 3 Department of Population Medicine and Lifestyle Diseases Prevention, Medical University of Bialystok, Waszyngtona 13 St, 15-269 Bialystok, Poland

**Keywords:** psoriasis, lichen planus, vitiligo, ANGPTL4, angiopoietin-like 4, coronary artery disease

## Abstract

Psoriasis, vitiligo and lichen planus (LP) are autoimmune skin diseases associated with metabolic syndrome. Angiopoietin-like 4 (ANGPTL4) is a member of angiopoietin-like proteins, which play an important role in lipid metabolism, and its serum concentration has been proposed as a biomarker of cardiometabolic complications, especially coronary artery disease (CAD). The study involved 56 patients with abovementioned dermatoses and 29 sex- and age-matched volunteers without dermatoses. ANGPTL4 serum concentration was measured by ELISA. ANGPTL4 concentration was statistically significantly higher in patients with LP compared to the control group (*p* < 0.01); moreover, it was significantly higher than in patients with psoriasis and vitiligo (*p* < 0.001, *p* < 0.01, respectively). There was no statistically significant difference in ANGPTL4 concentration between patients with psoriasis or vitiligo and controls. There was no correlation between ANGPTL4 concentration and age or BMI in all study groups. There was a positive correlation between ANGPTL4 concentration and fasting glucose (R = 0.43) and AST activity (R = 0.39) in psoriatic patients and ALT activity in patients with vitiligo (R = 0.44). ANGPTL4 could be a potential marker of metabolic complications in patients with LP, especially CAD. Perhaps patients with LP are more prone to CAD compared to the other two dermatoses, which requires further research.

## 1. Introduction

Psoriasis is one of the most common, chronic skin diseases. It occurs with a frequency of 2–4% on average worldwide [1]. It is genetically determined, but also modified, by environmental factors [2] and even despite extensive research on this matter, its pathogenesis is still not fully understood. Immunological disturbances are also key components of psoriasis pathology. Autoimmunity has been postulated, and specific autoantibodies have been recently detected [2,3]. There are many types of psoriasis, but the most frequent, which is plaque psoriasis, presents with erythematous-papular lesions and psoriatic plaques with a scaly surface [4].

Lichen planus (LP) is a dermatosis of uncertain but surely complex etiology, which occurs with unknown frequency, estimated to be slightly over 1% of the population [5]. It usually affects adults, with no gender predilection, but some sources report on higher prevalence in women than men [5]. Many factors are taken into account in the pathogenesis of LP. First, there is evidence of potential genetic predisposition for LP occurrence, since some gene polymorphisms of immune-, inflammation- and oxidative stress-related genes were detected [5]. Second, several factors have been proved to trigger LP lesions. These are, among others, hepatitis virus C (HCV) and other viral infections, stressful events or medications [5]. Last, but not least, LP is obviously associated with immune disturbances and is considered a T-cell-mediated autoimmune disorder [5]. LP involves especially glabrous skin, in approximately half of the patients, mucosal membranes and less frequently, nails or scalp. Its predominant manifestation is red-violaceus polygonal papules with a white shiny surface called Wickham’s striae [6].

Vitiligo is a skin disease of not fully understood etiology, which is characterized by the presence of well-defined depigmented macules caused by the loss or dysfunction of melanocytes [7]. Its frequency is estimated to be up to 4% of the population, with no sex predominance [7]. The onset may occur at any age, but vitiligo usually appears for the first time in children or young adults [7]. Vitiligo is classified into two types—segmental and non-segmental, of which the latter is predominant [7]. The pathogenesis of vitiligo includes genetic background, autoimmune mechanisms, oxidative stress, inflammation and melanocyte detachment mechanism [8].

**Figure 1:**
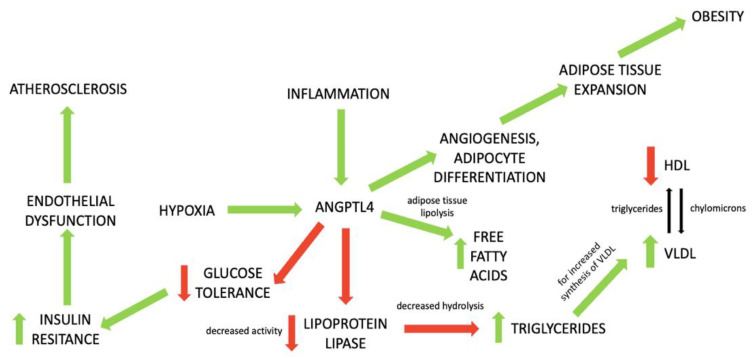
The role of ANGPTL4 in the development of metabolic disorders. Green arrows mean stimulation; red arrows mean inhibition of process.

Angiopoietin-like 4 (ANGPTL4) is a member of angiopoietin-like proteins, which play an important role in lipid metabolism. They regulate the function of lipoprotein lipase, which in turn is responsible for the hydrolysis of fatty acids from triglyceride-rich lipoproteins and management of their distribution to peripheral tissues [9]. ANGPTL4 is predominantly expressed in adipose tissue and is particularly engaged in the inhibition of lipoprotein lipase [9]. ANGPTL4 regulation is dependent on food intake (it is upregulated during fasting and downregulated during fed conditions) and oxygen supply (induced by hypoxia) [9]. Induction of ANGPTL4 inhibits LPL activity and increases circulating triglycerides concentration; hence, this protein exerts an influence on the development of metabolic disorders [9]. The role of ANGPTL4 in these processes is presented in Figure 1 [9,10,11,12].

As it happens, the aforementioned skin diseases are related to metabolic syndrome (MS). Obviously, the tightest association is the one with psoriasis, but LP and vitiligo have also been linked to MS [13,14,15]. Our team has been exploring these interrelations for years. In a recently published paper, we demonstrated that mucosal addressin cell adhesion molecule-1 (MAdCAM-1) might play a role as an inflammation indicator in psoriasis, but also exerts a beneficial impact on the lipid profile in LP [16].

The aim of our study was to investigate the role of ANGPTL4 in patients with selected dermatoses of autoimmune origin, additionally related to MS. To the best of our knowledge, this is the first research analyzing ANGPTL4 in psoriasis, lichen planus and vitiligo.

## 2. Materials and Methods

The participants were selected from the Bialystok+ Polish Longitudinal University Study, which started in 2016 and is supposed to enroll 10,000 randomly chosen participants in total. As of now, 56 patients (30 females and 26 males), 23 with plaque-type psoriasis, 15 with lichen planus (including several subjects from the Department of Dermatology of the same University) and 18 with vitiligo were enrolled in our study. They were all adults of Caucasian ethnicity originating from one city. They were compared with 29 sex- and age-matched volunteers without skin diseases. All participants signed informed written consents before initiation. Body mass index (BMI) was calculated as weight/height^2^(kg/m^2^). All subjects were subdivided into groups according to BMI: BMI 1 was related to normal-weight (BMI 18.5–24.9), second group—BMI 2—overweight (BMI 25–29.9) and BMI 3, obesity (BMI > 30). Laboratory tests including C-reactive protein (CRP), complete blood count (CBC), serum glucose, total cholesterol (Chol), triglycerides (TG) and asparagine and alanine aminotransferases (AST, ALT) were performed before treatment. The study was approved by the Bioethical Committee of Medical University in Bialystok, Bialystok, Poland (number: R-I-002/108/2016) and was in accordance with the principle of the Helsinki Declaration.

### 2.1. Serum Collection

Fasting blood samples were received from subjects and volunteers without dermatoses using vacutainer tubes with a clot activator. Samples were centrifuged at 2000× *g* for 10 min and preserved at −80 °C until analyses. ANGPTL4 concentrations were measured using an enzyme immunoassay kit supplied by Cloud Clone^®^ Houston, Texas, USA (SEB019Hu). Optical density was read at a wavelength of 450 nm. The concentrations were measured by interpolation from calibration curves prepared with standard samples supplied by the manufacturer. All the tests were performed by the same investigator in standardized laboratory settings.

### 2.2. Statistical Analysis

Normality of distribution was tested using the Shapiro–Wilk W test. The non-Gaussian data were presented as median (full range) and analyzed using the non-parametric Kruskal–Wallis test. The correlations were analyzed using Spearman’s Rank correlation analysis. Statistical analysis was conducted using the GraphPad Prism 9.20 software (GraphPad Prism 9.20 Software, USA). The differences were deemed statistically significant when *p* < 0.05.

## 3. Results

Basic characteristics of patients and controls are presented in Table 1. The study involved 56 patients with autoimmune dermatoses: 23 with plaque psoriasis, 15 with lichen planus and 18 with vitiligo, and 29 subjects without dermatoses who served as a control group. There was no significant difference between patients and controls in terms of age, gender or BMI (NS). Moreover, there were no significant differences between all four groups in terms of basic results of laboratory investigations (NS).

**Figure 2:**
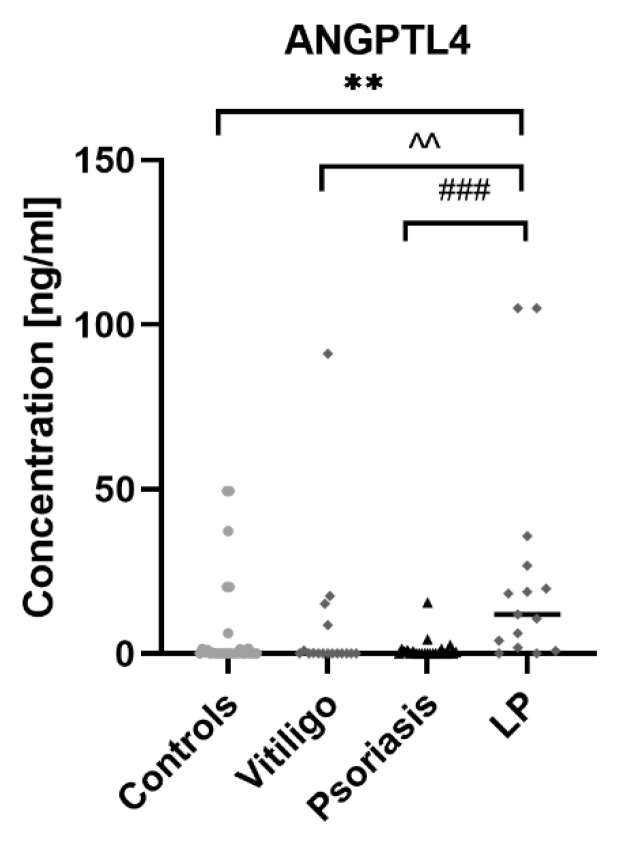
Concentrations of ANGPTL4 in patients with psoriasis, vitiligo and lichen planus, as well as in control group. ** means statistically significant difference between patients with LP and controls with *p* < 0.01; ^^ means statistically significant difference between patients with LP and vitiligo with *p* < 0.01; ### means statistically significant difference between patients with LP and psoriasis with *p* < 0.001.

Serum concentrations of ANGPTL4 in patients and controls are presented in Figure 2. (raw data in Table S1.).

**Figure 3:**
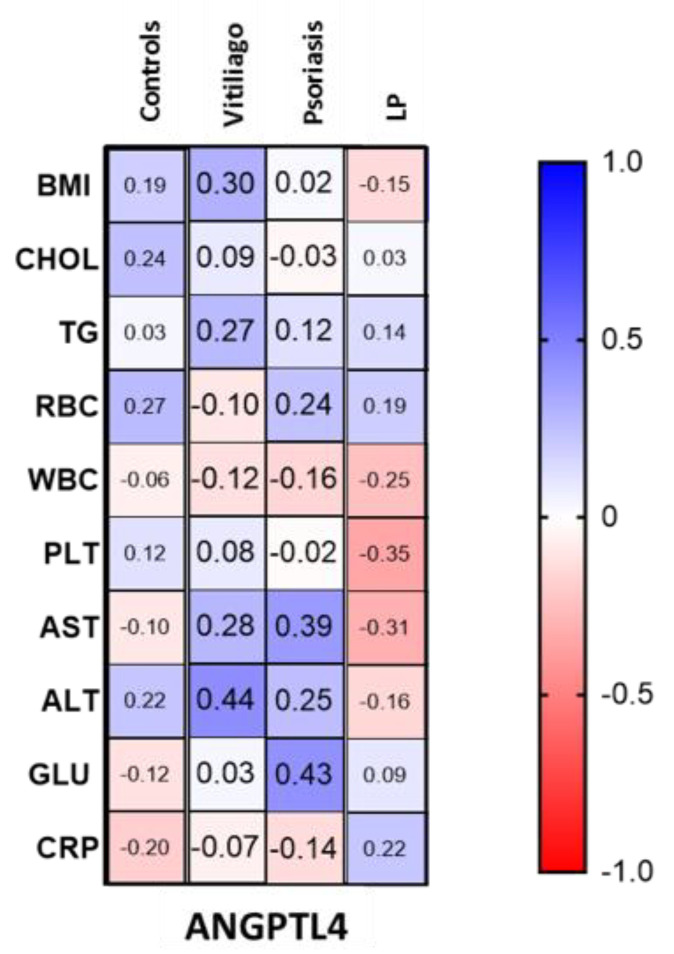
Correlations between ANGPTL4 concentrations and laboratory parameters in patients with vitiligo, psoriasis and lichen planus, as well as in controls.

ANGPTL4 concentration was statistically significantly higher in patients with LP compared to the control group (*p* < 0.01); moreover, it was significantly higher than in psoriatic patients (*p* < 0.001) and patients with vitiligo (*p* < 0.01). There was no statistically significant difference in ANGPTL4 concentration between patients with psoriasis and controls or in patients with vitiligo and controls (both NS). There was no correlation between ANGPTL4 concentration and age or BMI. Correlations between ANGPTL4 concentrations and laboratory parameters in particular groups of patients and controls are presented in Figure 3.

There was a positive correlation between ANGPTL4 concentration and fasting glucose (R = 0.43) and AST activity (R = 0.39) in patients with psoriasis. Moreover, there was a positive correlation between ANGPTL4 concentration and ALT activity in patients with vitiligo (R = 0.44). In the LP group, there was a downward trend for PLT and AST activity and ANGPTL4 concentration (R= −0.35, R= −0.31, respectively).

## 4. Discussion

Psoriasis is particularly well-related to metabolic disorders and many of these associations are bidirectional. Psoriatic patients are at an increased risk of cardiovascular disorders and have, therefore, a shorter life duration [13]. LP has also been described to be associated with MS [15]. In a big meta-analysis by Ying et al., it was determined that patients with LP have probably a 2-fold higher frequency of MS, which may be additionally dependent on its clinical type [15]. In recent years, publications regarding the connection between MS and vitiligo also appeared [14].

Research has shown the role of ANGPTL4 in the development of metabolic disorders based on observation of humans with loss-of-function mutations [17]. Such subjects tended to have elevated concentrations of HDL and decreased concentrations of triglyceride-rich lipoproteins [17]. Moreover, loss of ANGPTL4 leads to increased insulin sensitivity and improvement of glucose metabolism [9]. In the mouse model, it was observed that hepatocyte-specific depletion of *ANGPTL4* prevents diet-induced obesity, leads to a decrease in circulating triglycerides, increases insulin sensitivity and glucose tolerance and inhibits the progression of atherosclerosis [18]. Thus, it seems that increased ANGPTL4 blood concentration could be considered a marker of metabolic complications. Nevertheless, ANGPTL4 has been also reported to have two-faced properties in relation to atherosclerosis [9]. Plasma concentrations of ANGPTL4 have been proved to be a predictive factor for future cardiovascular events, but of contradictory nature. For instance, in one study by Muendlein et al., its increased concentration exacerbated the risk of coronary artery disease (CAD) [19], and in the other, by Sun et al., decreased ANGPTL4 was significantly related to CAD, regardless of lipid concentrations, and presented as a CAD biomarker [20]. Georgiadi et al. suggested in their study that ANGPTL4 has additional influence on atherosclerotic plaque progression, beyond the impact on plasma lipids. They postulated that ANGPTL4 is capable of slowing down the development of plaques by reducing the inflammatory response to saturated fats [21].

In our study, we observed a significantly higher concentration of ANGPTL4 in patients with LP compared to controls and two other groups with dermatoses. Since all of these diseases have been associated with MS, it seems that ANGPTL4 could become a marker of metabolic complications only in subjects with LP. Apparently, it should be investigated in larger groups to establish its definite application. Another conclusion that could be drawn from this observation is that, since ANGPTL4 has been established especially as a CAD marker, patients with LP are potentially more prone to CAD in particular, compared to the other two dermatoses.

As for the association with age and BMI of patients, the literature data suggest that they are positively correlated with ANGPTL4 concentrations [9], but we did not observe such a tendency in any of the studied groups.

Analyzing the relationship between the protein and laboratory parameters, we found a positive correlation between ANGPTL4 concentration and fasting glucose, but only in patients with psoriasis, which is consistent with the available literature [9]. Although such correlations have been proved, the actual role of ANGPTL4 in type 2 diabetes mellitus (DM) remains unclear due to inconsistent data from medical research—in some of them, patients with DM presented elevated ANGPTL4 serum concentrations and in the other, decreased [22]. Perhaps increased serum concentration of ANGPTL4 in patients with psoriasis could indicate an increased risk of carbohydrate metabolism disorders, but obviously, other factors must take part in this interplay considering the insignificant difference in ANGPTL4 concentration between such patients and controls. We found no correlations between ANGPTL4 and TGs, and the lack of such an evident relationship has been reported in the medical literature as well [9].

Moreover, there was a positive correlation between ANGPTL4 concentration and ALT activity in patients with vitiligo and AST activity in patients with psoriasis. In the LP group, there was a downward trend for PLT and AST activity and ANGPTL4 concentration. In the study on subjects with hepatic steatosis, Altun et al. observed no significant correlation between ANGPTL4 and ALT, but they did find a significant negative correlation with AST [23]. It should be further investigated in the future whether ANGPTL4 could become a marker of liver complications in subjects with vitiligo and psoriasis.

ANGPTL4 has been investigated as a therapeutic target, although there have been some obstacles in drug development [9]. So far, there have been no such medications finally introduced, but it could be interesting to investigate them in patients with LP in relation to the improvement of skin conditions.

The limitation of our study is that it involved subjects of only Caucasian ethnicity coming from one city. The number of participants is relatively low, although as this longitudinal study from which we selected our subjects is still going on, we expect this number to increase and to expand our investigation. As for this study, we used only one ELISA method.

## 5. Conclusions

This is the first study to report on the role of ANGPTL4 in patients with psoriasis, lichen planus and vitiligo. Although they are all associated with metabolic disorders, it seems that only in subjects with LP, ANGPTL4 could serve as a marker of metabolic complications, especially coronary artery disease. Perhaps patients with LP are more prone to coronary artery disease compared to the other two dermatoses, which requires further research.

## Figures and Tables

**Table 1:** Basic characteristics of patients and controls.

Parameter	Controls (*n* = 29)	Vitiligo (*n* = 18)	Psoriasis (*n* = 23)	LP (*n* = 15)
Sex (M/F)	15/14	9/9	11/12	6/9
Age [years]	46 (25–75)	53 (26–75) NS	45 (26–76) NS	58 (21–77) NS
BMI	27 ± 4	27 ± 5 NS	27 ± 4 NS	29 ± 5 NS

NS, non-significant; BMI, body mass index.

## Data Availability

Data available upon request.

## Supplementary Materials

The following are available online at https://www.mdpi.com/article/10.3390/metabo12090877/s1, Table S1. Raw data.
