# Supplementary material for: Angiopoietin-Like 4 (ANGPTL4) in Patients with Psoriasis, Lichen Planus and Vitiligo—A Pilot Study from the Bialystok+ Polish Longitudinal University Study
Source: Metabolites. 2022 Sep 17;12(9):877. doi: 10.3390/metabo12090877 (PMC9500972; doi:10.3390/metabo12090877)
Supplement: Supplementary file 1 [file metabolites-12-00877-s001.zip › metabolites-1808193-supplementary.pdf]

No	Group	ANGPTL4 concentration (ng/ml)
1	control	6,172
2	control	0,012
3	control	0,01
4	control	0,013
5	control	0,02
6	control	0,014
7	control	1,272
8	control	0,013
9	control	6,736
10	control	0,25
11	control	1,06
12	control	0,022
13	control	49,37
14	control	0,018
15	control	0,03
16	control	0,027
17	control	0,031
18	control	1,476
19	control	0,015
20	control	0,066
21	control	0,326
22	control	49,35
23	control	0
24	control	20,261
25	control	20,283
26	control	0,039
27	control	0,027
28	control	1,61
29	control	37,144
30	vitiligo	0,012
31	vitiligo	0,019
32	vitiligo	0,892
33	vitiligo	0,022
34	vitiligo	0,017
35	vitiligo	0,023
36	vitiligo	0,04
37	vitiligo	0,132
38	vitiligo	0,17
39	vitiligo	0,018
40	vitiligo	8,688
41	vitiligo	0,01
42	vitiligo	15,076
43	vitiligo	0,16
44	vitiligo	17,532
45	vitiligo	7,849
46	vitiligo	0,014
47	vitiligo	91,074
48	psoriasis	0,017

49	psoriasis	0,016
50	psoriasis	15,49
51	psoriasis	0,024
52	psoriasis	0,012
53	psoriasis	1,432
54	psoriasis	1,054
55	psoriasis	0,65
56	psoriasis	4,28
57	psoriasis	0,037
58	psoriasis	0,022
59	psoriasis	0,019
60	psoriasis	2,572
61	psoriasis	0,022
62	psoriasis	0,021
63	psoriasis	0,476
64	psoriasis	1,394
65	psoriasis	0,612
66	psoriasis	0,025
67	psoriasis	0,023
68	psoriasis	0,017
69	psoriasis	0,019
70	psoriasis	0,016
71	lichen planus	104,922
72	lichen planus	0,024
73	lichen planus	0,033
74	lichen planus	10,6
75	lichen planus	6,178
76	lichen planus	1,748
77	lichen planus	11,888
78	lichen planus	18,738
79	lichen planus	35,632
80	lichen planus	19,688
81	lichen planus	0,83
82	lichen planus	3,93
83	lichen planus	104,922
84	lichen planus	18,25
85	lichen planus	26,69
